# Molecular Regulation of Differential Lipid Molecule Accumulation in the Intramuscular Fat and Abdominal Fat of Chickens

**DOI:** 10.3390/genes14071457

**Published:** 2023-07-17

**Authors:** Jingjing Li, Qinke Huang, Chaowu Yang, Chunlin Yu, Zengrong Zhang, Meiying Chen, Peng Ren, Mohan Qiu

**Affiliations:** 1 School of Life Science and Engineering, Southwest University of Science and Technology, Mianyang 621010, China; jingjingyi11@126.com (J.L.);; 2 Guangyuan City Animal Husbandry Seed Management Station, Guangyuan 628107, China; 3 Sichuan Animal Science Academy, Chengdu 610066, China

**Keywords:** chicken, abdominal fat, intramuscular fat, integration analysis, glycerophospholipid

## Abstract

Reducing abdominal fat (AF) accumulation and increasing the level of intramuscular fat (IMF) simultaneously is a major breeding goal in the poultry industry. To explore the different molecular mechanisms underlying AF and IMF, gene expression profiles in the breast muscle (BM) and AF from three chicken breeds were analyzed. A total of 4737 shared DEGs were identified between BM and AF, of which 2602 DEGs were upregulated and 2135 DEGs were downregulated in the BM groups compared with the AF groups. DEGs involved in glycerophospholipid metabolism and glycerolipid metabolism were potential regulators, resulting in the difference in lipid metabolite accumulation between IMF and AF. The PPAR signaling pathway was the most important pathway involved in tissue-specific lipid deposition. Correlation analysis showed that most representative DEGs enriched in the PPAR signaling pathway, such as FABP5, PPARG, ACOX1, and GK2, were negatively correlated with PUFA-enriched glycerophospholipid molecules. Most DEGs related to glycerophospholipid metabolism, such as GPD2, GPD1, PEMT, CRLS1, and GBGT1, were positively correlated with glycerophospholipid molecules, especially DHA- and arachidonic acid (ARA)-containing glycerophospholipid molecules. This study elucidated the molecular mechanism underlying tissue-specific lipid deposition and poultry meat quality.

## 1. Introduction

Lipids are one of the major components of the chicken body. Intensive selection for a rapid growth rate leads to excessive abdominal fat (AF) accumulation [1], which exerts a negative impact on consumer acceptance and health [2]. However, intramuscular fat (IMF) is a major component that produces meat flavor, and the presence of intramuscular fat improves meat tenderness and juiciness [3,4,5]. To meet consumers’ needs, reducing AF accumulation and increasing the levels of IMF simultaneously has become a major breeding goal in the poultry industry.

A previous study verified that a desirable broiler line with higher IMF and lower AF could be obtained using genetic selection [6]. AF-derived preadipocytes have higher adipogenic differentiation ability than IMF-derived preadipocytes [7]. Large transcriptomic differences were identified between IMF- and AF-derived preadipocyte differentiation [7]. These results indicated that the deposition of AF and IMF are subject to different regulatory mechanisms in chickens. Although transcriptomics has been applied to reveal the molecular mechanisms underlying AF and IMF deposition between different chicken breeds [8,9], it is more effective to find a balanced selection between AF and IMF, rather than selection for higher IMF or lower AF alone. Therefore, systematic and deep research is needed to reveal the different molecular mechanisms underlying AF and IMF deposition.

Previous work found that adipose tissues from different locations display unique biochemical characteristics [10]. The major lipid class in AF was triglyceride [1], whereas phospholipid was the major lipid class in IMF [11]. The fatty acid composition of AF was also significantly different from that in IMF [11]. In our recently published study, liquid chromatography (LC)–mass spectrometry (MS)-based lipidomics was used to characterize the comprehensive lipid composition of the abdominal region and breast muscle. A total of 209 lipid molecules were commonly determined as marker candidates for AF and IMF among three chicken breeds [12]. Varying lipid molecule compositions of adipose tissue and muscle have profound effects on meat quality. For example, the fatty acid composition determines the firmness/oiliness of adipose tissue and the oxidative stability of muscle, which in turn affects flavor and muscle color [11]. Phospholipids contain a high proportion (above 30%) of essential polyunsaturated fatty acids (PUFAs), which are more susceptible to oxidation and, therefore, are important for meat flavor development [13]. In addition to serving as substrates for volatile flavor, docosahexaenoic acid (DHA) is an n-3 PUFA that is essential in human biochemical reactions [14]. The significantly higher PUFA-enriched glycerophospholipid metabolites, especially DHA-enriched glycerophospholipid metabolites, such as PC (18:3e/19:2), PC (18:4e/21:2), PC (22:6e/17:2), PE (18:2e/22:6), and PE (18:2e/22:5), elucidated the underlying reasons why IMF could improve meat flavor and nutritional value [12]. However, further investigation is required to combine these lipid metabolites with the underlying molecular mechanisms that regulate and optimize body fat distribution in chickens.

In the current study, RNA sequencing (RNA-seq) was used to explore the regulatory genes and signaling pathways involved in AF and IMF deposition. To identify differentially expressed genes (DEGs) between AF and IMF more accurately, we selected three native chicken breeds (the Guangyuan grey chicken, Jiuyuan black chicken, and Tibetan chicken) for analysis. We conducted an integrative analysis of transcriptome and previous lipidomics data from the three chicken breeds and then identified the common correlations between DEGs and differential metabolites. These results can help to identify valuable gene markers for specific lipid metabolite accumulation in the abdominal region and muscle.

## 2. Materials and Methods

### 2.1. Animals and Sample Collection

We obtained 300-day-old Guangyuan grey chickens, Jiuyuan black chickens, and Tibetan chickens from Sichuan Tianguan Ecological Agriculture and Animal Husbandry Co., Ltd. (Guangyuan City, Sichuan Province), Wanyuan Hengkang Agricultural Development Co., Ltd. (Wanyuan City, Sichuan Province), and Maoxian Jiuding Ecological Poultry Breeding Co., Ltd. (Wenchuan City, Sichuan Province), respectively. All chickens were kept under similar environmental and feeding conditions. Room temperatures from 15 to 20 °C were maintained using controlled ventilation and heating. The diet for all chickens, according to the feeding standard <<NY/T33-2004>>, is shown in Table S1. Nine female chickens with normal and similar body weights from each breed were selected for sample collection. After a 12 h fast and breathing anesthesia until unconscious, the chickens were sacrificed by cutting the carotid artery. AF and breast muscle (BM) were collected and immediately stored at −80 °C until RNA isolation was performed.

### 2.2. Transcriptome Profiling

RNA from 54 tissue samples was extracted, as described previously [15]. RNA purity was checked using a NanoPhotometer spectrophotometer (Thermo Scientific, Waltham, MA, USA). RNA integrity was assessed using an Agilent 2100 Bioanalyzer (Agilent Technologies, Santa Clara, CA, USA) and agarose gel electrophoresis. The RNA integrity numbers (RINs) for the 54 samples were all greater than 6.0, and the RNA in each sample exceeded 400 ng. Qualified RNA from 3 BM and 3 AF samples collected from each chicken breed were pooled into two separate mixtures, respectively. A total of 18 mixture samples (JYBM1, JYBM2, JYBM3, JYAF1, JYAF2, JYAF3, GYBM1, GYBM2, GYBM3, GYAF1, GYAF2, GYAF3, TCBM1, TCBM2, TCBM3, TCAF1, TCAF2, TCAF3) were obtained for further analysis. The library preparations were sequenced using an Illumina Novaseq platform, and 150 bp paired-end reads were generated. Adapter sequences and other low-quality data were removed using Trimmomatic v0.32. Sequencing reads were then aligned to the chicken reference genome of GRCg6a using HISAT2 v2.0.5.

### 2.3. Differential Expression Analysis and KEGG Enrichment Analysis

Feature Counts v1.5.0-p3 was used to count the read numbers mapped to each gene. Then, the FPKM of each gene was calculated based on the length of a gene and the read count mapped to that gene. Differential expression of transcripts was performed using the DESeq2 R package (v 1.20.2). The resulting *p*-values were adjusted using the Benjamini and Hochberg’s approach for controlling the false discovery rate (FDR). Genes with an adjusted *p* (Padj)-value < 0.05 and |log2(Fold Change)| ≥ 1 were considered DEGs. A Kyoto Encyclopedia of Genes and Genomes (KEGG) pathway enrichment analysis was performed using the KEGG online website (http://www.genome.jp/kegg/ (accessed on 6 November 2022)). We used the clusterProfiler R package to test the statistical enrichment of differentially expressed genes in the KEGG pathway. The pathways with an adjusted *p*-value less than 0.05 were considered significantly enriched.

### 2.4. cDNA Synthesis and Real-Time qRT-PCR Analysis

The PrimeScript™ RT Reagent Kit was used for the reverse transcription of total RNA. Primers used for quantitative real-time (qRT-PCR) were listed in Table 1. The qRT-PCR was conducted with a CFX-96 (Bio-Rad, Inc., Richmond, CA, USA) qRT-PCR system using a 10 µL reaction volume with 1 µL of cDNA, 0.5 µL of the forward and reverse primers (10 µM) for each gene, 5 µL of TB Green Premix Ex Taq (Tli RNase H Plus) (TaKaRa), and 3 µL of double-distilled H_2_O. All mRNA expression levels were normalized to the GAPDH mRNA level. The relative expressions of related genes were calculated using the 2^−∆∆Ct^ method, and three biological replicates were performed on each sample. Pearson’s correlation coefficients were generated to evaluate the accuracy of RNA-Seq.

### 2.5. Lipid Extraction and Lipidomic Analysis

Lipid extraction from the 54 tissue samples was performed according to a previous study [12]. UHPLC-MS/MS analyses were performed using a UHPLC system (Thermo Fisher, Waltham, MA, USA) coupled with an Orbitrap Q ExactiveTM HF mass spectrometer (Thermo Fisher). Samples were injected into a Thermo Accucore C30 column (150 × 2.1 mm, 2.6 μm) at a flow rate of 350 μL/min. Mobile phase buffer A was acetonitrile/water (6/4) with 10 mM ammonium acetate and 0.1% formic acid, whereas buffer B was acetonitrile/ isopropanol (1/9) with 10 mM ammonium acetate and 0.1% formic acid. The solvent gradient was set as follows: 30% B, initial; 30% B, 2 min; 43% B, 5 min; 55% B, 5.1 min; 70% B, 11 min; 99% B, 16 min; and 30% B, 18.1 min. A Q ExactiveTM HF mass spectrometer was operated in positive and negative polarity modes under the following conditions: sheath gas, 20 arbitrary units; sweep gas, 1 arbitrary unit; auxiliary gas rate: 5 (7 in negative polarity modes); spray voltage, 3000 V; capillary temperature, 350 °C; heater temperature, 400 °C; S-lens radio frequency, 50 Hz; scan range, 114–1700 m/z; normalized collision energy: 25; 30 (20; 24; 28 in negative polarity modes); and injection time, 100 ms.

The raw data files were processed using Compound Discoverer 3.1 (Thermo Fisher) to perform peak alignment, peak picking, and quantitation of each metabolite. Then, the peak intensities were normalized to total spectral intensity according to the set parameters. The main parameters were set as follows: retention time tolerance, 0.2 min; actual mass tolerance, 5 ppm; signal intensity tolerance, 30%; signal/noise ratio, 3; and minimum intensity, 100,000. The normalized data were used to predict the molecular formula based on additive ions, molecular ion peaks, and fragment ions. Then, the peaks were matched with Lipidmaps and Lipidblast databases to obtain accurate qualitative and relative quantitative results.

### 2.6. Integrated Analysis of Transcriptomics and Lipidomics

In our recently published study, we used the same samples for lipidomic analysis. A total of 209 lipid molecules were commonly determined as the maker candidates for AF and IMF among the three chicken breeds. Based on those results, 3 breast muscle samples and 3 abdominal fat samples were pooled into two separate mixtures, respectively, when we conducted transcriptomics analysis. We also calculated the mean of the relative abundance for the corresponding samples (3 breast muscle or 3 abdominal fat samples) of lipid molecules to guarantee that the absolute same samples were used for integrated analysis of transcriptomics and lipidomics. Pair-wise Pearson’s correlation coefficients were generated to evaluate the correlation between differential metabolites and DEGs between AF and BM in Guangyuan grey chickens, Jiuyuan black chickens, and Tibetan chickens, respectively. Correlation coefficients were visualized with heatmap plots using the R package “complex_heat_map”.

## 3. Results

### 3.1. Overall Description of Sequencing Data

Overall, we obtained 118.9 Gb clean bases and 792,671,934 clean reads from RNA-seq data. These clean reads were uniquely mapped to the chicken genome (GRCg6a), and the mapping frequencies were found to vary from 86.91% to 91.27% (Table S2). Among the mapped reads, an average of 92.95% of the total mapped reads was mapped to exons, 3.30% was mapped to introns, and 3.75% was mapped to the intergenic regions.

### 3.2. Identification of DEGs between AF and BM

**Figure 1:**
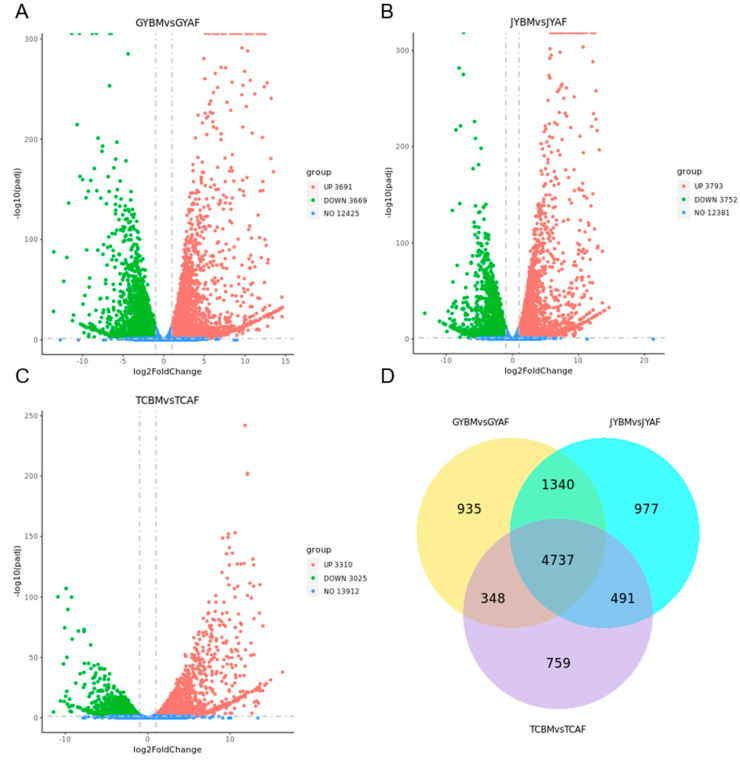
(**A**–**C**) Volcano plot showing DEGs in GYBM vs. GYAF, JYBM vs. JYAF, TCBM vs. TCAF. Red dots represent significantly up-regulated genes and green dots represent significantly down-regulated genes. (**D**) Venn diagrams showing differentially expressed genes in the BM and AF of Guangyuan grey chickens, Jiuyuan black chickens, and Tibetan chickens.

Based on the criterion of “Padj < 0.05 and |log2(Fold Change)| ≥ 1”, a total of 7360 known DEGs were screened in the breast muscle of Guangyuan grey chickens (GYBM) vs. the abdominal fat of Guangyuan grey chickens (GYAF); 7545 known DEGs were screened in the breast muscle of Jiuyuan black chickens (JYBM) vs. the abdominal fat of Jiuyuan black chickens (JYAF); and 6335 known DEGS were screened in the breast muscle of Tibetan chickens (TCBM) vs. the abdominal fat of Tibetan chickens (TCAF) (Figure 1A–C). Among these DEGs, 4737 genes were identified as common DEGs between the BM and AF groups in Guangyuan grey chickens, Jiuyuan black chickens, and Tibetan chickens (Figure 1D). Although the values of log2 (fold change) identified differences between the three chicken breeds, these 4737 shared DEGs showed a completely consistent trend between BM and AF. We found that 2602 shared DEGs were upregulated and 2135 shared DEGs were downregulated in the BM group compared with the AF group.

As expected, we observed that large numbers of genes or transcription factors involved in lipid metabolism were differentially expressed. In Table 2, we list the shared DEGs related to glycerolipid metabolism, glycerophospholipid metabolism, and sphingolipid metabolism. We found that 21 upregulated DEGs were enriched in glycerophospholipid metabolism pathway including glycerol-3-phosphate dehydrogenase 2 (GPD2), acetylcholinesterase (ACHE), glycerol-3-phosphate dehydrogenase 1 (GPD1), phospholipase A2 group IVB (PLA2G4B), phospholipase A2 group IVE-like 2 (PLA2G4EL2), phospholipase A2 group IIE (PLA2G2E), phospholipase A2 group IIA (PLA2G2A), protein interacting with cyclin A1 (PROCA1), phosphatidylserine synthase 2 (PTDSS2), phosphatidylethanolamine N-methyltransferase (PEMT), cardiolipin synthase 1 (CRLS1), phosphoethanolamine/phosphocholine phosphatase 1 (PHOSPHO1), ethanolamine-phosphate phospho-lyase (ETNPPL), glyceronephosphate O-acyltransferase (GNPAT), ST6 N-acetylgalactosaminide α-2,6-sialyltransferase 4 (ST6GALNAC4), globoside α-1,3-N-acetylgalactosaminyltransferase 1 (GBGT1), α-1,4-galactosyltransferase (A4GALT), LOC101750362, ST3 β-galactoside α-2,3-sialyltransferase 6 (ST3GAL6), phosphate cytidylyltransferase 1A (PCYT1A), and phosphate cytidylyltransferase 1B (PCYT1B). However, more DEGs related to glycerolipid metabolism had a significantly higher expression in the AF groups, including phospholipid phosphatase 5 (PLPP5), phospholipid phosphatase 4 (PLPP4), 1-acylglycerol-3-phosphate O-acyltransferase 2 (AGPAT2), 1-acylglycerol-3-phosphate O-acyltransferase 4 (AGPAT4), LOC422609, lipin 2 (LPIN2), diacylglycerol kinase theta (DGKQ), patatin-like phospholipase domain containing 2 (PNPLA2), diacylglycerol O-acyltransferase 2 (DGAT2), patatin-like phospholipase domain containing 3 (PNPLA3), acyl-CoA wax alcohol acyltransferase 1 (AWAT1), glycerol kinase 2 (GK2), lipoprotein lipase (LPL), aldehyde dehydrogenase 3 family member A2 (ALDH3A2), LOC425137, and aldo-keto reductase family 1 member E2 (AKR1E2). Sphingolipid metabolism-related DEGs were significantly higher in AF groups, including neuraminidase 3 (NEU3), arylsulfatase A (ARSA), sphingosine phosphate lyase 1 (SGPL1), sphingomyelin phosphodiesterase 2 (SMPD2), ceramide synthase 4 (CERS4), and N-acylsphingosine amidohydrolase 1 (ASAH1). These genes would be the key potential regulators resulting in the difference between lipid metabolite accumulation in the abdominal region and muscle.

### 3.3. KEGG Enrichment Analysis of DEGs Involved in Lipid Metabolism

**Figure 2:**
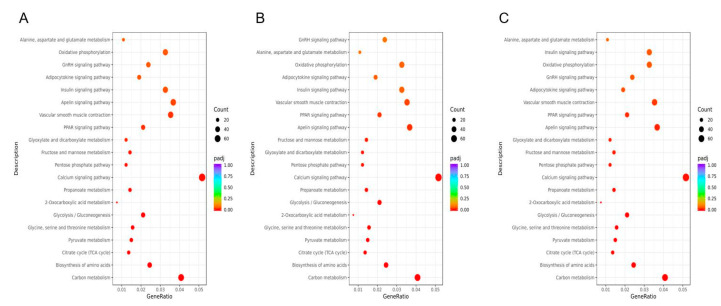

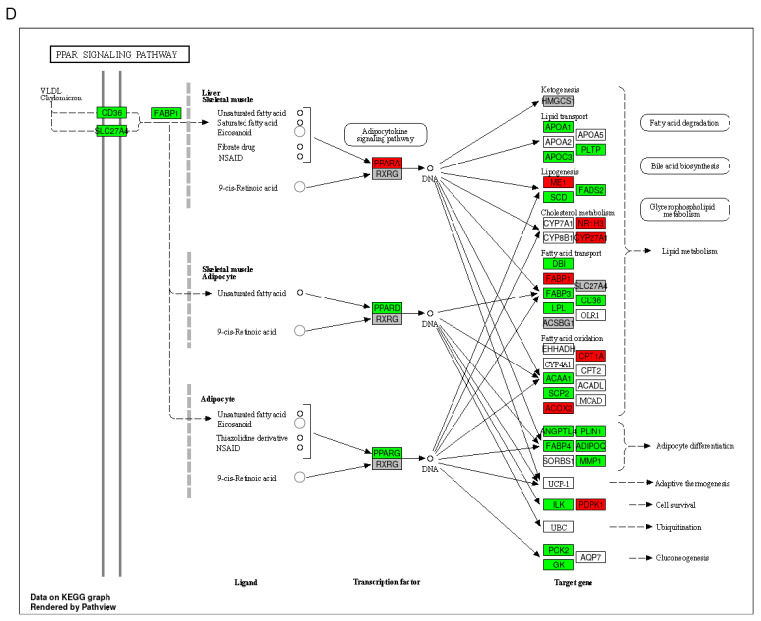
KEGG pathway enrichment analysis of the DEGs between BM and AF in Guangyuan grey chickens (**A**), Jiuyuan black chickens (**B**), and Tibetan chickens (**C**). (**D**) PPAR signaling pathway plot. The node color indicates the expression of genes: (red) up-regulated and (green) down-regulated in the BM groups relative to the AF groups. GeneRatio: the ratio of the number of differential genes annotated to the KEGG pathway number to the total number of differential genes.

To eliminate the effect of breed on lipid metabolism, we used 4737 common DEGs simultaneously identified in the three chicken breeds for functional enrichment analysis. Here, we display the top 20 pathways for the GYBM vs. GYAF, JYBM vs. JYAF, and TCBM vs. TCAF comparisons. Based on 4737 shared DEGs, Figure 2A–C shows the results for KEGG enrichment in Guangyuan grey chickens, Jiuyuan black chickens, and Tibetan chickens, respectively. Interestingly, we found that these shared DEGs identified in the GYBM vs. GYAF, JYBM vs. JYAF, and TCBM vs. TCAF comparisons were all enriched in 15 KEGG pathways (*p* < 0.05), including carbon metabolism; biosynthesis of amino acids; the citrate cycle (TCA cycle); pyruvate metabolism; glycine, serine, and threonine metabolism; 2-oxocarboxylic acid metabolism; glycolysis/gluconeogenesis; propanoate metabolism; the calcium signaling pathway; the pentose phosphate pathway; glyoxylate and dicarboxylate metabolism; fructose and mannose metabolism; the apelin signaling pathway; the peroxisome proliferator-activated receptor (PPAR) signaling pathway; and vascular smooth muscle contraction (*p* < 0.05). Thirty-one shared DEGs were significantly enriched in the PPAR signaling pathway, which was the sole pathway involved in lipid metabolism (Figure 2D). In Guangyuan grey chickens and Tibetan chickens, 28 DEGs were significantly enriched in the adipocytokine signaling pathway (*p* < 0.05), while no significant difference was observed in Jiuyuan black chickens (*p* > 0.05).

### 3.4. Gene Expression Validation of DEGs using qRT-PCR

**Figure 3:**
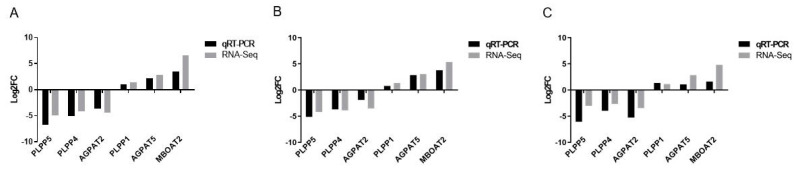
Results of the qRT-PCR validation in (**A**) GYBM vs. GYAF (**B**) JYBM vs. JYAF and (**C**) TCBM vs. TCAF.

The results from the RNA-Seq analysis were further confirmed by validating the expression data for six randomly selected DEGs. The log2 (fold change) of RNA-Seq data for each gene was the average value, so we also calculated the average value of the qRT-PCR result. The accuracy of the RNA-Seq was authenticated by the consistency in the qRT-PCR results using the RNA-Seq data with a correlation coefficient of 0.94 (Figure 3).

### 3.5. Correlations between Shared DEGs and Differential Lipid Molecules

**Figure 4:**
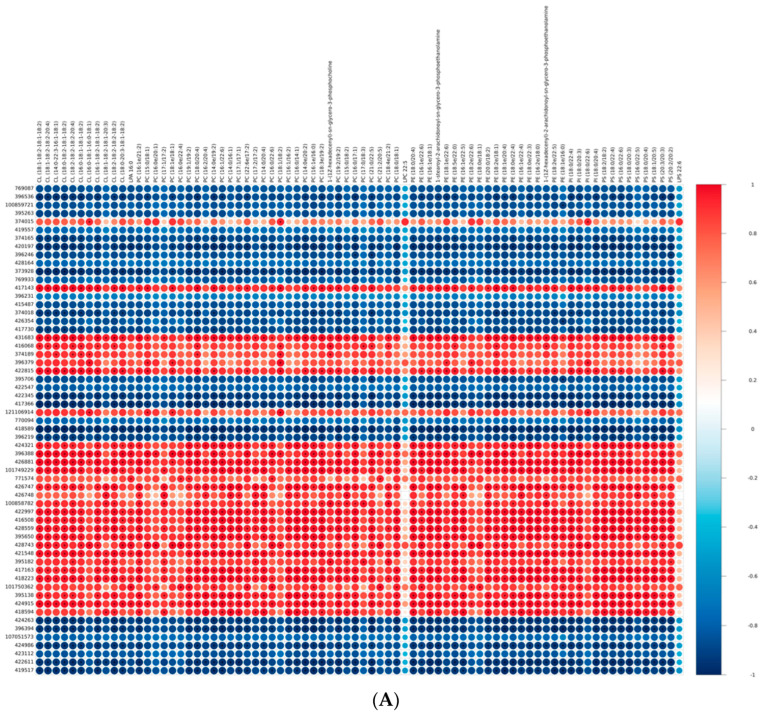

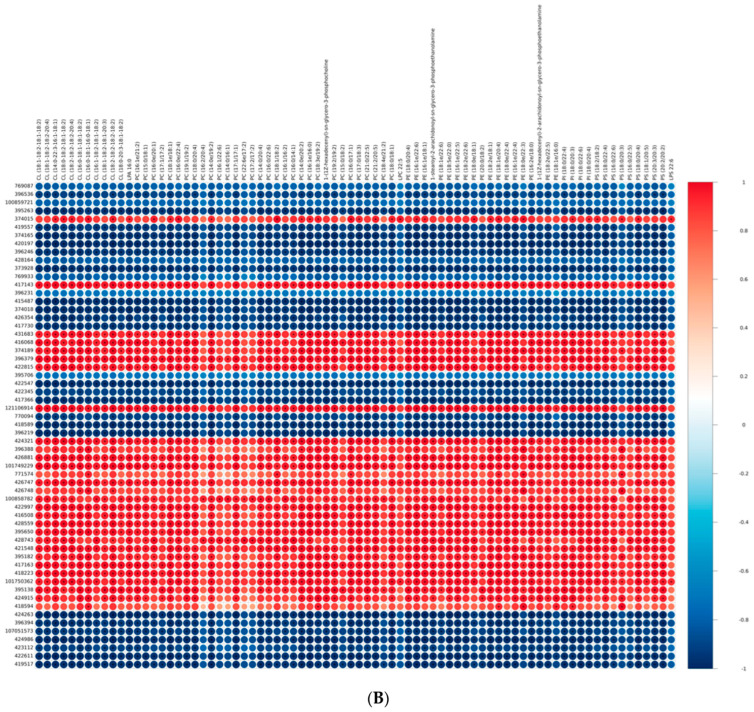

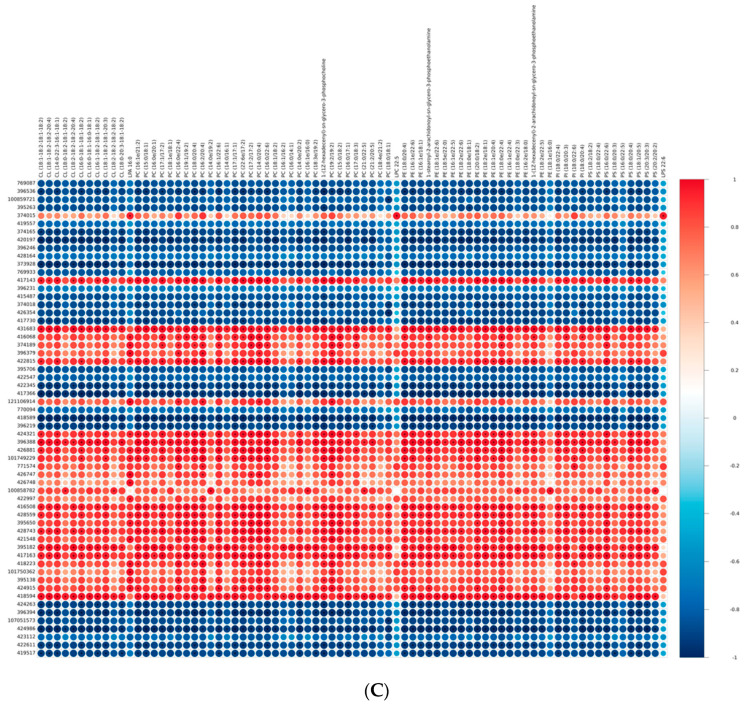
Integration analysis of lipidomics and transcriptome profiles for (**A**) GYBM vs. GYAF, (**B**) JYBM vs. JYAF, and (**C**) TCBM vs. TCAF. Each row represents a gene, and each line represents a lipid molecule. * *p* < 0.01.

**Figure 5:**
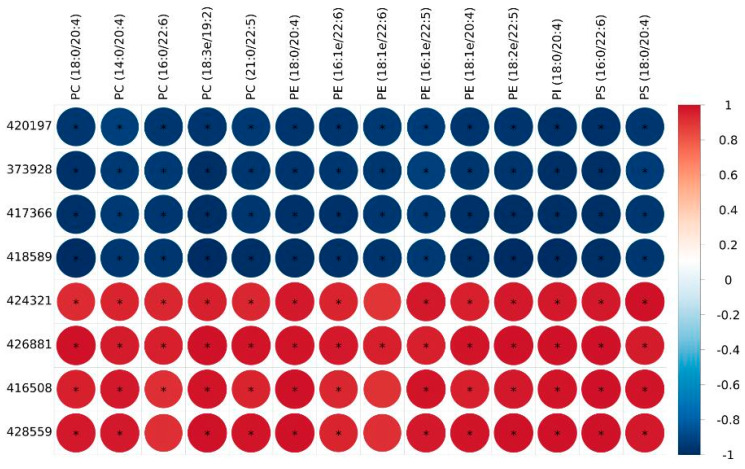
Significant shared correlations between important PUFA-enriched glycerophospholipid molecules and genes among the three chicken breeds. * *p* < 0.01. The y-axis is the gene id.

In our recently published study, the same samples were used for lipidomic analysis. We identified a large number of shared glycerophospholipid lipid molecules that were significantly upregulated in IMF compared with those in AF. These glycerophospholipid lipid molecules included 11 cardiolipin (CL), 1 phosphatidic acid (PA), 33 phosphatidylcholines (PC), 19 phosphatidylethanolamines (PE), 4 phosphatidylinositols (PI), and 10 phosphatidylserines (PS). The difference between these individual glycerophospholipid compounds was the root cause of the higher content of phospholipids in intramuscular fat. In the present study, the shared DEGs related to glycerophospholipid metabolism and the PPAR signaling pathway were screened. It is reasonable to speculate that these DEGs were involved in the biosynthesis of glycerophospholipid molecules in BM. Therefore, an integrated analysis of transcriptomics and lipidomics was conducted to evaluate the correlation between differential glycerophospholipid metabolites and DEGs related to glycerophospholipid metabolism and the PPAR signaling pathway. In total, 59 shared DEGs (31 related to the PPAR signaling pathway and 28 related to glycerophospholipid metabolism) and 78 shared differential glycerophospholipid metabolites were used in the correlation analysis for Guangyuan grey chickens, Jiuyuan black chickens, and Tibetan chickens, respectively. The heatmap plots are presented in Figure 4. Each row represents a glycerophospholipid molecule, and each line represents a gene. A total of 1896, 3034, and 1496 significant correlations between DEGs and differential metabolites were identified in Guangyuan grey chickens, Jiuyuan black chickens, and Tibetan chickens, respectively. In order to identify the candidate genes for specific lipid molecule accumulation more accurately, shared significant correlations among the three chicken breeds were screened. A total of 777 significant shared correlations were detected. Considering the important role of PUFA in meat flavor and nutritional value, we concentrated on the PUFA-enriched glycerophospholipid molecules (Figure 5). We found most representative DEGs enriched in the PPAR signaling pathways were negatively correlated with PUFA-enriched glycerophospholipid molecules (*p* < 0.01). For example, the mRNA expressions of FABP5 (gene id 420197), PPARG (gene id 373928), ACOX1 (gene id 417366), and GK2 (gene id 418589) were simultaneously negatively correlated with PC (18:3e/19:2), PE (18:2e/22:5), PC (18:0/20:4), PE (18:0/20:4), and PE (18:1e/20:4) (*p* < 0.01). Considering the important role of eicosapentaenoic acid (EPA) and docosahexaenoic acid (DHA) in humans, we found that these four genes also significantly inhibited the biosynthesis of glycerophospholipid molecules carrying DHA and EPA, such as PC (16:0/22:6), PE (18:1e/22:6), PE (16:1e/22:6), PS (16:0/22:6), PC (21:0/22:5), PE (16:1e/22:5), and PE (18:2e/22:5) (*p* < 0.01). However, most DEGs related to glycerophospholipid metabolism were positively correlated with glycerophospholipid molecules, especially DHA- and arachidonic acid (ARA)-containing glycerophospholipid molecules. For example, GPD2 (gene id 424321), GPD1 (gene id 426881), PEMT (gene id 416508), and CRLS1 (gene id 428559) were collectively involved in the positive regulation of ARA-enriched metabolites, including PC (18:0/20:4), →PC (14:0/20:4), PE (18:0/20:4), PE (18:1e/20:4), PI (18:0/20:4), and PS (18:0/20:4) (*p* < 0.01). Furthermore, GPD1 and GDP2 were positively related to PC (16:0/22:6), PE (16:1e/22:6), and PS (16:0/22:6) (*p* < 0.01).

In our previous study, we identified that two triacylglycerol metabolites (TAG (16:1-18:1-18:1) and TG (16:1(9Z)/16:1(9Z)/18:1(9Z))[iso3])) were significantly higher in AF tissue, which might be a possible reason for the higher triglyceride content verified in AF. To decrease AF content, we also conducted a correlation analysis between two triacylglycerol molecules and the shared DEGs involved in glycerolipid metabolism and the PPAR signaling pathway. Unfortunately, no shared correlations were identified among the three chicken breeds (*p* > 0.01).

## 4. Discussion

In poultry, excessive AF accumulation causes low slaughter efficiency and exerts a negative impact on consumer acceptance and health [2]. Increased IMF content contributes to better meat quality, including tenderness, flavor, and nutritional value [4].

Lowering the AF content and increasing the IMF content can effectively increase the economic value of broilers, which has become a major breeding goal in the poultry industry. Most previous studies concentrated on exploring the potential gene markers regulating AF or IMF deposition unilaterally based on a comparison of two types of chicken breeds whose AF deposition or IMF deposition was exceptionally varied. For example, after obtaining gene expression profiles of breast muscles for Beijing-You and AA chickens, researchers identified candidate genes related to IMF deposition [8]. A previous study also used extremely high- and low-abdominal fat chicken groups to investigate crucial genes related to AF deposition [16]. Although the genetic mechanisms underlying chicken fat deposition have been widely studied, few studies were conducted to determine the mechanism that leads to tissue-specific lipid molecule accumulation. Nowadays, indigenous chicken breeds are preferred by customers due to their better meat quality. Previous studies indicated that the intramuscular fat content in these breeds is higher than that in commercial chicken breeds [8]. In this study, we selected three indigenous chicken breeds for integrative analysis of transcriptome and previous lipidomics data.

Unlike the marbling distribution of IMF in domestic animals, the IMF of chickens cannot be separated visually. To elucidate the regulatory mechanism underlying tissue-specific lipid metabolism, DEGs were identified between AF and BM. Those genes related to glycerolipid metabolism, glycerophospholipid metabolism, and sphingolipid metabolism were displayed. To eliminate the effects of breed, three chicken breeds (the Guangyuan grey chicken, Jiuyuan black chicken, and Tibetan chicken) were selected in this study. Only the shared DEGs identified in GYBM vs. GYAF, JYBM vs. JYAF, and TCBM vs. TCAF simultaneously were used for subsequent analysis. A total of 4737 shared DEGs showed a completely consistent trend between BM and AF, of which 2602 DEGs were upregulated and 2135 DEGs were downregulated in the BM group compared with the AF group. We found that more shared DEGs related to glycerophospholipid metabolism were upregulated in the BM groups, while the expressions of more DEGs related to glycerolipid metabolism were higher in the AF groups. Interestingly, a previous study indicated that phospholipids were found in a considerable proportion of BM [11], while the triglyceride content in AF was significantly higher than that in BM [17]. Thus, these DEGs were potential regulators causing the difference in triglyceride and phospholipid content between AF and BM. It is well known that the AF content is far greater than the IMF content in chickens. At the cellular level, researchers have indicated that the lipogenesis of preadipocytes derived from AF was significantly increased compared with that of preadipocytes derived from BM [18]. Consistent with previous results in vitro, the downregulated DEGs related to glycerolipid metabolism were responsible for the decrease in total lipids in BM.

Based on 4737 shared DEGs, the results of the KEGG signaling pathway analysis showed that DEGs between BM and AF were jointly enriched in 15 pathways. Thirty-one shared DEGs were significantly enriched in the PPAR signaling pathway with the classic mediation of lipid metabolism. Among these 31 DEGs, the RNA-seq data showed that the PLIN1, FABP4, SCD, PPARG, GK2, CD36, APOC3, FABP7, LPL, RXRG, SLC27A6, FABP3, ACSL1, DBI, ACOX1, PLTP, APOA1, ANGPTL4, FABP5, ACSL4, ILK, ACAA1, PCK2, and SLC27A2 genes were downregulated, while only 7 genes (CYP27A1, HMGCS1, ME1, LOC121106914, RXRA, ACOX2, and FABP1) were upregulated in the BM group. Many studies have confirmed the role of these genes in lipid metabolism [19,20,21]. For example, PLIN1, which was reported to protect lipid droplets from the hydrolytic activity of hormone-sensitive lipase [18], had significantly higher expression levels in AF. PPARG, the most adipocyte-specific member of the PPAR family, is mainly expressed in adipose tissue [20]. Fatty acid-binding proteins (FABPs), also known as intracellular lipid chaperones, are a family that regulates lipid trafficking and responses in cells [19]. Fatty acids cross the cell membrane via a protein-mediated mechanism, and FABPs are responsible for fatty acid uptake [22]. Different isoforms of the FABP family are uniquely expressed in tissues involved in lipid metabolism [23]. The mRNA expressions of heart FABP (H-FABP/FABP3), adipocyte FABP (A-FABP/FABP4), and epidermal FABP (E-FABP/FABP5), brain FABP (B-FABP/FABP7) were significantly higher in AF, while the expression of liver FABP (L-FABP/FABP1) was significantly higher in IMF. This indicated that FABPs may provide tissue-specific control of lipid metabolism. Consistent with previous results in vitro [18], it was considered that tissue-specific lipid deposition mostly depended on the changed expression of related genes through the PPAR pathway.

Glycerophospholipids are dominant in cell membranes and play a vital role in cellular functions such as signal transduction, protein function, and regulation of transport processes [24]. In our recently published study, lipidomic analysis showed that a significantly higher abundance of 11 CL, 1 PA, 33 PC, 19 PE, 4 PI, and 10 PS was observed in IMF compared with that in AF. PUFAs are predominantly deposited in the glycerophospholipid of muscle rather than free fatty acids [25,26]. The higher abundance of PUFA-containing glycerophospholipids in IMF could elucidate its beneficial roles in imparting a characteristic flavor and nutritional value to meat [12]. Therefore, exploring the important candidate gene markers for specific PUFA-containing glycerophospholipid molecules would contribute to a more thorough understanding of IMF deposition and poultry meat quality.

The limited amounts of n-3 PUFAs present in meats are nutritionally important [27]. It is worthwhile to mention the beneficial function of n-3 PUFAs, notably EPA and DHA. They play a vital role in the prevention and treatment of certain diseases, such as Alzheimer’s disease, cardiovascular disease (CVD), type 2 diabetes, hypertension, psychiatric diseases, and several cancers [28,29]. Furthermore, DHA is the core nutrient for human brain function, neuronal development, and visual acuity [30]. In the present study, significantly negative correlations were observed between four genes (FABP5, PPARG, ACOX1, and GK2) and seven glycerophospholipid molecules carrying DHA and EPA (PC (16:0/22:6), PE (18:1e/22:6), PE (16:1e/22:6), PS (16:0/22:6), PC (21:0/22:5), PE (16:1e/22:5), and PE (18:2e/22:5)). However, previous studies have shown that DHA and EPA could be natural PPAR agonists to activate PPARG signaling [31,32]. In golden pompano hepatocytes, the overexpression of PPARG increased the DHA content, whereas the suppression of PPARG expression diminished this effect, suggesting that PPARG plays an active role in regulating DHA content [33]. Here, we conducted a correlation analysis between PPARG and individual DHA- and EPA-containing glycerophospholipid rather than total DHA or EPA content. The molecular mechanism underlying the PPARG regulation of these seven glycerophospholipid molecules carrying DHA and EPA needs to be verified in chicken intramuscular preadipocytes. Similar to DHA, ARA also has a significant physiological function during development and growth, especially for the central nervous system and retina [34]. In this study, GPD2, GPD1, PEMT, CRLS1, and GBGT1 were collectively involved in the positive regulation of ARA-enriched metabolites. Whether these DEGs positively regulated ARA content or these ARA-enriched molecules also needs to be verified at the cellular level.

## 5. Conclusions

In this study, an analysis of transcriptome data identified 4737 shared differentially expressed genes between IMF and AF deposition among three chicken breeds. Differentially expressed genes involved in glycerophospholipid metabolism and glycerolipid metabolism were potential regulators resulting in the difference between lipid metabolite accumulation in IMF and AF. The PPAR signaling pathway was the most important pathway involved in tissue-specific lipid deposition. Most representative DEGs enriched in the PPAR signaling pathways, such as FABP5, PPARG, ACOX1, and GK2, were negatively correlated with PUFA-enriched glycerophospholipid molecules. Most DEGs related to glycerophospholipid metabolism, such as GPD2, GPD1, PEMT, CRLS1, and GBGT1, were positively correlated with glycerophospholipid molecules, especially DHA- and ARA-containing glycerophospholipid molecules. These results contributed to a more thorough understanding of lipid deposition and poultry meat quality.

## Figures and Tables

**Table 1:** The specific primers for qRT-PCR used in this study.

Accession Number	Gene Symbol	Primer Sequence	Product Size
XM_040689357.2	PLPP5	F: GCGCCGATGTTCTTCATTGC R: TTGGTGAAGACCCCGTTGAG	143
XM_040674888.2	PLPP4	F: CTTCGGGGTCTTCGTTTTTACAG R: CCGCCGGATTATTTTCACCAC	181
XM_040685441.2	AGPAT2	F: TGCTTTGGTGCAGGCTTGTG	122
		R: GACCACGGTTTTGATGATCCTGG	
XM_424730.8	PLPP1	F: CCCCTTCCAGAGAGGAGTTTTC R: GCAAGGAAGTGAGGACGCAG	300
XM_419916.8	AGPAT5	F: ACTCCATGCGCTACTTCCTG R: GGTAGATGGTGTAGAGGCGG	147
NM_001031090.2	MBOAT2	F: GCCACCAGCACTACGGG R: AACCAAATAGCCGCAAGCAA	119

**Table 2:** Common DEFs related to glycerolipid and glycerophospholipid metabolism and between the BM and AF groups for three chicken breeds.

Gene ID	Gene Name	Putative Function in KEGG Pathway	Log2 (Fold Change)			Trend	Padj
			GYBM vs. GYAF	JYBM vs. JYAF	TCBM vs. TCAF		
427138	PLPP1	Glycerolipid metabolism	1.40	1.36	1.14	Up	
421898	AGPAT5		2.86	3.05	2.85		
421925	MBOAT2		6.58	5.34	4.81		<0.01
418121	AGK		1.87	1.51	1.64		
426846	LIPG		3.86	3.42	3.72		
424321	GPD2	Glycerophospholipid metabolism	1.82	2.54	2.84	Up	<0.01
396388	ACHE		5.09	5.51	4.65		
426881	GPD1		9.02	10.24	8.94		
101749229	PLA2G4B		4.00	3.03	2.62		
771574	PLA2G4EL2		4.62	2.33	3.17		
426747	PLA2G2E		3.20	3.37	3.05		
426748	PLA2G2A		5.19	4.80	4.46		
100858782	PROCA1		2.18	2.62	4.53		
422997	PTDSS2		2.19	2.29	1.35		
416508	PEMT		1.77	2.09	1.85		
428559	CRLS1		1.62	1.74	1.91		
395650	PHOSPHO1		4.10	3.92	3.65		
428743	ETNPPL		3.46	2.85	5.20		
421548	GNPAT		2.28	2.22	2.37		
395182	ST6GALNAC4		1.16	1.21	1.56		
417163	GBGT1		2.94	2.86	2.63		
418223	A4GALT		2.62	2.30	2.18		
101750362	LOC101750362		6.63	7.24	6.42		
395138	ST3GAL6		2.10	2.27	1.43		
424915	PCYT1A		1.50	1.55	1.65		
418594	PCYT1B		3.68	6.62	3.41		
428318	CERS4L	Sphingolipid metabolism	2.41	2.34	3.05	Up	<0.01
422529	SGMS2		4.82	4.12	3.51		
770752	PLPP5	Glycerolipid metabolism	−4.89	−4.21	−3.02	Down	<0.01
428987	PLPP4		−4.20	−3.91	−2.68		
772114	AGPAT2		−4.42	−3.54	−3.48		
421578	AGPAT4		−2.88	−2.03	−2.15		
422609	LOC422609		−1.06	−1.21	−1.14		
421059	LPIN2		−1.93	−1.56	−1.32		
427381	DGKQ		−1.13	−1.30	−1.13		
431066	PNPLA2		−6.44	−5.28	−4.78		
421309	DGAT2		−5.06	−2.44	−3.23		
418233	PNPLA3		−2.59	−3.61	−3.05		
428693	AWAT1		−3.71	−2.91	−2.01		
418589	GK2		−6.34	−5.37	−4.89		
396219	LPL		−4.74	−4.73	−4.99		
417615	ALDH3A2		−2.90	−3.11	−2.46		
425137	LOC425137		−2.41	−2.45	−2.06		
418171	AKR1E2		−2.13	−2.09	−1.79		
424263	GPD1L2	Glycerophospholipid metabolism	−11.69	−8.63	−9.70	Down	<0.01
396394	PLA2G4A		−2.28	−1.61	−1.42		
107051573	PLA2G3		−5.74	−6.12	−5.52		
424986	PLD1		−1.56	−1.68	−1.83		
423112	CHKA		−1.49	−2.11	−1.22		
422611	CDS1		−3.99	−4.12	−4.07		
419517	CDS2		−4.31	−3.15	−2.78		
430542	NEU3	Sphingolipid metabolism	−1.11	−1.69	−1.49	Down	<0.01
426863	ARSA		−2.99	−2.33	−2.32		
423714	SGPL1		−2.64	−2.00	−2.52		
770663	SMPD2		−1.22	−1.25	−1.34		
420050	CERS4		−1.89	−1.58	−1.10		
422727	ASAH1		−3.15	−2.28	−2.26		

## Data Availability

The RNA-seq raw data supporting the conclusions of this article have been deposited into Sequence Read Archive under accession number PRJNA902098.

## Supplementary Materials

The following supporting information can be downloaded at: https://www.mdpi.com/article/10.3390/genes14071457/s1, Table S1: Composition of diets distributed to the three chicken breeds during the rearing period; Table S2: Overview of the sequencing data.
