# Supplementary material for: Molecular Regulation of Differential Lipid Molecule Accumulation in the Intramuscular Fat and Abdominal Fat of Chickens
Source: Genes (Basel). 2023 Jul 17;14(7):1457. doi: 10.3390/genes14071457 (PMC10379444; doi:10.3390/genes14071457)
Supplement: Supplementary file 1 [file genes-14-01457-s001.zip › genes-2458415-supplementary.pdf]

Table S1. Composition of diets distributed during the rearing period for three chicken breeds.

Ingredient (g/100g)	Starter (1-42d)	Grower (42-91d)	Finisher (92-300d)
Corn	61.5	63.6	70.00
Soybean meal	26.00	21.00	14.00
Fish meal	5.00	4.00	3.00
Wheat	-	3.00	4.50
Rice bran	4.00	3.00	4.50
Soybean oil	-	2.00	1.00
Calcium hydrogen phosphate	1.00	1.00	0.80
Calcium carbonate	1.20	1.10	1.00
Salt	0.30	0.30	0.20
Premix feed	1.00	1.00	1.00
**Nutrient composition**			
Metabolic energy (MJ/kg)	11.88	12.34	12.30
Crude protein (%)	19.40	17.20	14.40
Calcium (%)	1.02	0.92	0.76
Non-phytic acid phosphor (%)	0.44	0.41	0.35
Lysine (%)	1.06	0.90	0.71
Methionine + Cystine (%)	0.63	0.56	0.48

Table S2. Overview of sequencing data.

Sample ID	Raw reads	Clean reads	Clean bases (Gb)	Q20 (%)	Q30 (%)	Total map (%)
JYBM_1	41673704	40654372	6.1	98.07	94.89	88.15%
JYBM_2	45545020	44508856	6.68	98.09	94.94	88.14%
JYBM_3	46780550	45747200	6.86	98.08	94.93	87.92%
JYAF_1	48944482	47602366	7.14	97.18	93.31	88.61%
JYAF_2	44347290	43298328	6.49	97.47	93.9	88.95%
JYAF_3	45775342	44809226	6.72	97.2	93.33	89.01%
TCBM_1	45241964	44292770	6.64	97.75	94.13	87.54%
TCBM_2	46100068	45088468	6.76	98.06	94.9	86.91%
TCBM_3	41815008	41119492	6.17	98.15	95.05	89.39%
TCAF_1	41761752	40886366	6.13	97.52	93.9	90.81%
TCAF_2	44112166	43329870	6.5	97.4	93.5	91.27%
TCAF_3	46397890	45341888	6.8	97.29	93.5	89.07%
GYBM_1	44961136	43989802	6.6	97.72	93.96	87.88%
GYBM_2	44544188	43619268	6.54	97.57	93.6	87.69%
GYBM_3	41754904	40775508	6.12	98.06	94.88	88.34%
GYAF_1	49534854	48439594	7.27	97.12	93.02	89.53%
GYAF_2	48386116	47265004	7.09	97.73	94.37	90.76%
GYAF_3	42681110	41903556	6.29	97.56	93.92	90.82%
